# Prediction and control of COVID-19 spreading based on a hybrid intelligent model

**DOI:** 10.1371/journal.pone.0246360

**Published:** 2021-02-11

**Authors:** Gengpei Zhang, Xiongding Liu

**Affiliations:** 1 China Three Gorges University, Yichang, Hubei, China; 2 School of Automation Science and Engineering, South China University of Technology, Guangzhou, Guangdong, China; Texas A&M University College Station, UNITED STATES

## Abstract

The coronavirus (COVID-19) is a highly infectious disease that emerged in the late December 2019 in Wuhan, China. It caused a worldwide outbreak and a major threat to global health. It is important to design prediction and control strategies to restrain its exploding. In this study, a hybrid intelligent model is proposed to simulate the spreading of COVID-19. First, considering the effect of control measures, such as government investment, media publicity, medical treatment, and law enforcement in epidemic spreading. Then, the infection rates are optimized by genetic algorithm (GA) and a modified susceptible-infected-quarantined-recovered (SIQR) epidemic spreading model is proposed. In addition, the long short-term memory (LSTM) is imbedded into the SIQR model to design the hybrid intelligent model to further optimize other parameters of the system model, which can obtain the optimal predictive model and control measures. Simulation results show that the proposed hybrid intelligence algorithm has good predictive ability. This study provide a reliable model to predict cases of infection and death, and reasonable suggestion to control COVID-19.

## Introduction

In the past six months, Chinese people have been on a high level of containment due to the outbreak of the coronavirus 2019 throughout China [1]. However, due to the convenient of global transportation, the virus has rapidly spread around the world. Coronavirus was first discovered in Wuhan. At the early stage, people lack of knowledge about the virus and scarcity of medical resources, people were not aware of the virus and neglected to control the virus in its early stages, until the official announced that the disease can spread from person to person on January 20, 2020 [2]. Then, the government took a series of measures to prevent the spread of disease on January 21, like lockdown in Wuhan. Subsequently, all cities in China lockdown and cities across countries sealing off. Governments took many measures, such as closing public places, broadcasting propaganda, isolating people in their own homes, which lead to increase the awareness of self-protection for people. After three months of fighting against the epidemic, it has been successful controlled. However, the situation of the world is not optimistic, over 40 million people diagnosed in worldwide. The total number of deaths is over 1.2 million. Countries such as the United States, Brazil, Russia, and India, the pandemic is still quite severe today [3, 4]. Therefore, each countries and states should adopt prevention and control strategies to restrain the epidemic spreading. Currently, it is very important to establish and analyze disease-spreading model to predict disease development trends in order to prevent and control the spread of COVID-19.

Due to the outbreak of disease, vaccines and medicine treatments for the disease are still being researched, non-drug treatment becomes the main strategies to slow down the spread of the disease. The purpose of most of these prediction and control measures are reducing the probability of infection spreading during direct or indirect contact [5]. For example, paying attention to personal hygiene and wearing a mask, keeping social distance, and closing some public space such as schools and workplaces, in order to decrease the probability of propagation from person to person. Other measures like cutting off the way of transmission of diseases, such as disinfection in public places, improving the level of sanitation in public places and so on. All measures can alleviate the propagation of the epidemic [6–9]. How to quantify the impact of various prevention and control strategies on disease spreading is of great significance to guide disease control.

A common method utilizes mathematical modelling to describe the spreading dynamics of infectious diseases, like Ebola, SARS. It can accurately describe the spread of disease among individuals in theoretical framework to guide the development of the prevention and control measures [10–16]. There are many researchers studied epidemic spreading that have proposed some epidemic model and obtained some meaningful results. Most of researches based on complex networks, the classical epidemic models like susceptible-infected (SI), susceptible-infected-recovered (SIR), susceptible-exposed-infected-recovered (SEIR), susceptible-infected-quarantined-recovered-susceptible (SIQRS) [17–22]. Most of these models are applicable to describe disease spreading with a long incubation period, such as COVID-19. Based on data-driven, a modified SEIR model was proposed to analyze and predict COVID-19 spread [23]. Considering the effect of quarantine, B. K. Mishra et.al have proposed three quarantine models to analyze COVID-19 spreading [24]. Other recent studies, some researchers have considered the effects of the basic reproductive number, international conveyance, and some stochastic factors based regression models to predict and control the spreading of COVID-19 [25, 26]. However, traditional epidemic spreading models consider that all infected individual have the same infection rate, and the prediction of disease development trend has certain limitations. Although data-driven disease spreading models can accurately describe infection rates, the impact of government prevention and control measures on infection rates has not been quantitatively in detail. These measures, such as the laws, medical supplies, media coverage and investment, can reduce the spread of the disease. It is necessary to rationally arrange the optimal prevention and control strategies with limited resources to minimize the death rate.

To solve this problem, the GA and ANN [27–36] hybrid method is proposed to optimize epidemic dynamics model and predict the COVID-19 spreading. Genetic algorithm is an adaptive global optimization search algorithm formed by simulating the genetic and evolutionary process of biological species in natural environment [37]. Combining the viewpoint of biogenetics and realizing the improvement of individual adaptability through the mechanism of natural selection, heredity, and variation. Artificial intelligence (AI) is considered one of the most successful achievements of computer science, simulating the behavior of the human brain in data analysis. One of the AI branches is the artificial neural network (ANN). The information spreading process can simulate like communication between brain neurons and become a tool for analyzing complex and real systems [38]. In recent years, ANN models have been used to overcome the difficulties presented by health issues.

This contribution of this article is how to quantify the impact of the government’s prevention and control measures on epidemic infection rate, then obtaining the optimal model of disease spreading and the effective prevention and control strategy. In this article, based on the proposed SIQR model, the hybrid artificial neural network (ANN) model embedded genetic algorithm to predicting the spreading of COVID-19. It introduces an important prediction and control strategies that can give a guidance to the government. The data consistency is reduced due to the fluctuation of virus detection capability in a sudden change on February 12 and 13. In this paper, it is assumed that virus transmission in the environment is classified as human-to-human transmission. As a developing country, Brazil shows the epidemic data with periodicity and consistency, and then this article takes the Brazil data as analytic target. The simulation results based on the epidemic data of Brazil show that the proposed hybrid model could provide a basis for estimating the law of virus spread, and achieve accurate and robust performance. Moreover, the prediction results of a hybrid ANN-GN model is fit with the actual trend of epidemic development, which demonstrates that the openness, transparency, and efficiency of data releasing. Furthermore, this method can be extended to other countries if the actual data can be obtained.

The remainder of this article is organized as follows. Section “A hybrid model of COVID-19 spreading dynamics” introduces the framework of the proposed hybrid epidemic spreading model. Section “Methods” explains the method of GA and ANN to predict epidemic spreading. Section “Simulation results and discussion” provides the simulation results based on the epidemic data of Brazil and gives some discussion. The conclusions are provided at last.

### A hybrid model of COVID-19 spreading dynamics

**Fig 1:**
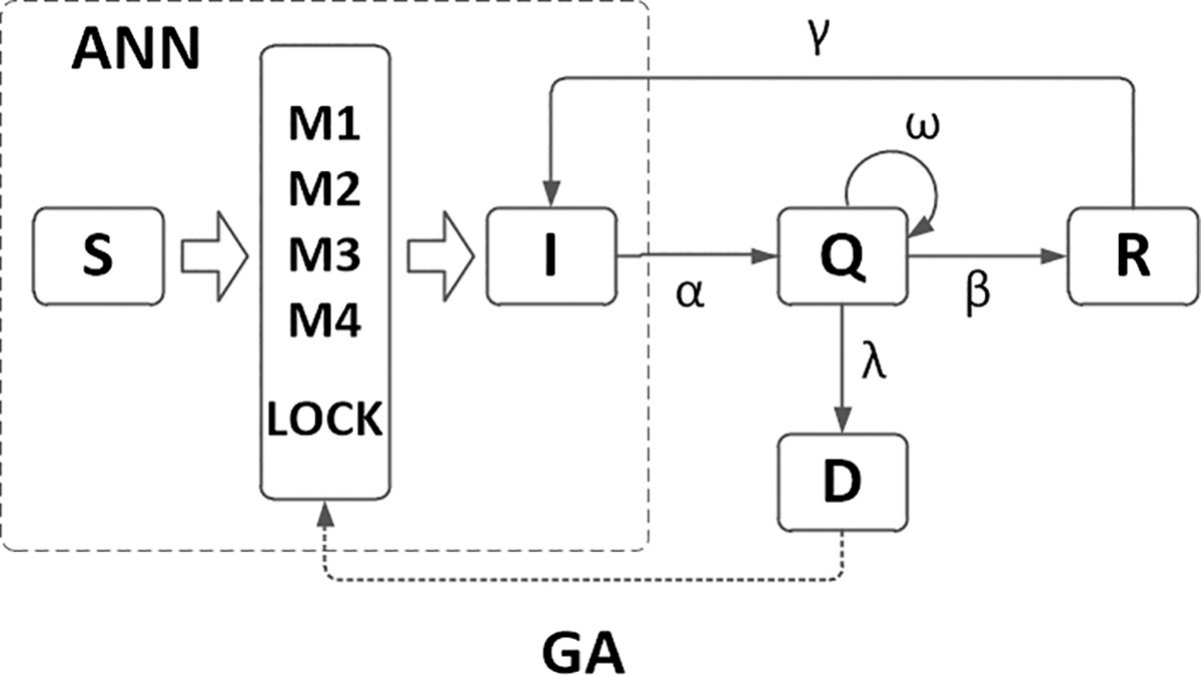
The flow diagram of the SIQR model.

In this section, we establish a mathematical model on COVID- 19 based on some assumptions. There are some literatures proposed models mainly based on real clinical data, predict and control measures. In this paper, we modified the previous model proposed in [18], and then extend the model structure by designing different infection rate. The model network diagram and the interaction individual components demonstrated in Fig 1. Every individual in network can only exist one of four independent states, namely, susceptible, infected, quarantined and recovered. For simply, it can be denoted by S, I, Q, and R, respectively. Each link represents the transformation relationship between nodes. Here, infected individuals include symptomatic infected individuals and asymptomatic infected individuals. Susceptible individual is infected with probability m (M1, M2, M3 and M4) if it is connected to an infected individual. Infective individuals are quarantined with probability α. In the process of quarantine, the asymptomatic infected individuals turn into symptomatic infected individuals with the probability ω. Quarantined individuals are treated with drugs that move into the recovered individuals with probability β. Some recovered individuals will relapse into infection due to their weakened immunity with probability γ. The probability of death during quarantine is λ. Here, setting a switch of city lockdown by the death rate, infection probability m is different based on the city situation (lockdown or not). The lockdown infection probability m is much lower under the strict government regulation.

According to the epidemic spreading, the dynamic equations can be written as follows:

$$\{\begin{array}{l} \frac{dS_{k}(t)}{dt}=-mS_{k}(t)\\ \frac{dI_{k}(t)}{dt}=mS_{k}(t)+\gamma R_{k}(t)-\alpha I_{k}(t)\\ \frac{dQ_{k}(t)}{dt}=\alpha I_{k}(t)-\beta Q_{k}(t)-\lambda Q_{k}(t)\\ \frac{dR_{k}(t)}{dt}=\beta Q_{k}(t)-\gamma R_{k}(t) \end{array}$$ (1)

Using the normalization condition, the probability of death can be obtained:

$$D=\frac{\lambda \alpha m\gamma }{\alpha \gamma (\lambda +m)+m\gamma ((\lambda +\beta )+\alpha \beta m}$$ (2)

In this paper, we consider the death rate as a very important factor to study the epidemic spreading. It not only depicts the fatality rate of the disease in the process of transmission, but also reflects the effectiveness of prevention and control measures. For example, money invested by the government can improve the medical level and effectively cut off the transmission route of the virus. Increasing publicity and awareness of prevention and control will also reduce the risk of infection. Law enforcement can also affect the spread of diseases, such as city lockdown and home quarantined. This paper argues that due to the strict control and quarantined measures taken by the government during the epidemic, the infected cases cannot infect susceptible people after quarantined. However, asymptomatic infected persons also have some ability to propagate the disease, and it is difficult to detect. Therefore, there is a certain relationship between the number of newly infected cases on Day t and the number of infected cases in the past k days. Moreover, the infection rate of patients is closely related to the time of infection. Since government measures can inhibit the spread of the disease, the infection rate of newly infected cases may vary from time to time on t day over the past k days. Further analyzing this difference and assigning different weights to different measures, we quantified the contribution of different measures to the infection rate at time t in newly infected cases. Then, the weighted accumulation was used to estimate the infection rate to establish the relevant epidemic prediction and control modeling.

**Fig 2:**
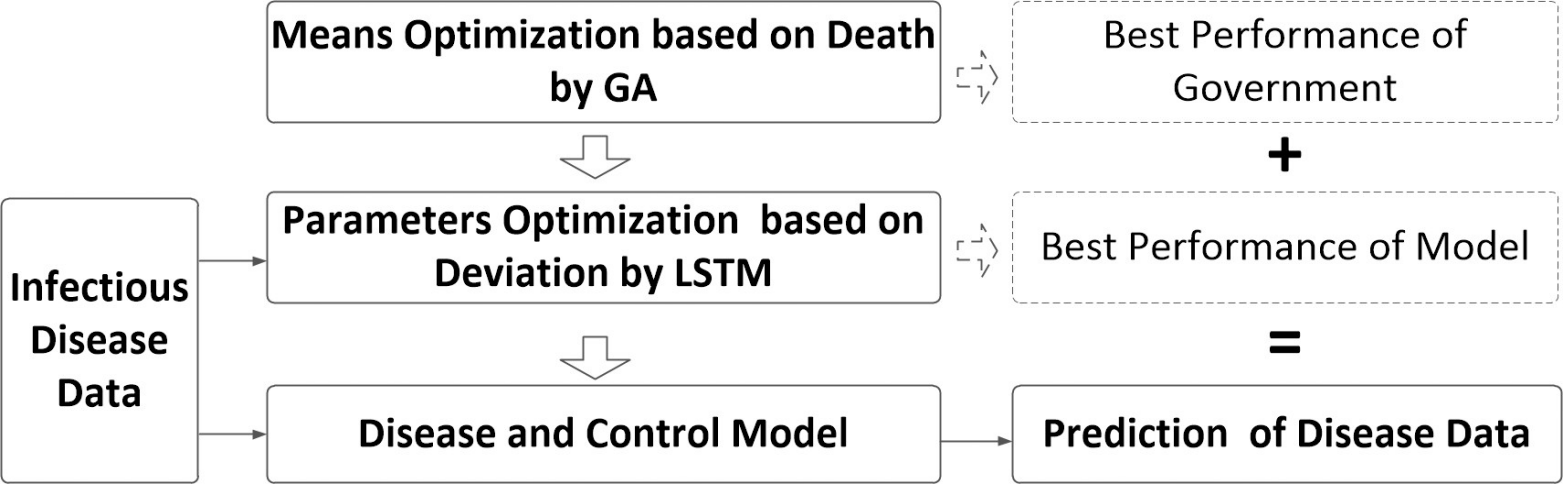
The hybrid model for COVID-19 prediction.

In addition, in order to study the relationship between the prediction and control measures with infection rate of the SIQR epidemic spreading model, the method of GA and LSTM are used to optimize the parameters of the spreading model. This paper considers the relationship between the rate of disease transmission and the measures taken by the government against the disease. The main factors include government investment, medical level, media publicity and law enforcement. Firstly, genetic algorithm is used to estimate the infection rate of the model, taking the acceptable mortality rate as the fitness function, and taking it as a basic basis for city lockdown parameters. At the same time, GA is further used to obtain the optimal means of government control by taking the minimum mortality rate as fitness function. The mutation law of GA is based on the interaction of four government measures. Furthermore, the infection rate bias and mortality bias were estimated through the LSTM network, the number of infected people was estimated by combining with the SIQR model, and the relevant parameters of the model were modified to obtain the best transmission model and predict the spread of the disease. By combining these two approaches, the optimal model of disease spreading and the optimal prevention and control strategies can be obtained. The number of infected and death cases based on the spreading model and development trend can be predicted. The framework of GA and ANN is shown in Fig 2.

## Methods

Epidemiological investigation and modeling are efficient tools to study the spread of epidemic diseases and offer the strategies to prevent and control of public health events with global impact, such as SARS, MERS and H1N1 influenza. Many researchers have achieved some results in novel coronavirus research using epidemiology and modeling analysis. The study of J. Chan et al. [39] found the first evidence of human-machine transmission. Then, some researchers combined with the analysis of some early cases and gave the mean incubation period and mean infection cycle of novel coronavirus. Traditional epidemic spreading models shows that the number of new infections cases is related to the number of infected individual and susceptible individual, but these models lack in-depth analysis of model parameters. In the process of disease transmission, the implementation of different control measures has a great impact on the prevention and control. For example, the government’s implement the disease prevention and control strategies, the formulation of relevant laws and regulations, financial investment and other measures can affect the spread of disease. The study found significant differences in the rates of infection among people of different ages. The main purpose of this paper is to study the impact of government measures on the spread of disease and to minimize the mortality rate, and to consider the impact of different age groups on the spread of disease, finally, we can obtain the optimized measures and the best performance model to prediction, as showing in Fig 2.

Unlike the traditional SIQR epidemic spreading model, which consists of invariable infected rate to describe the probability of infection. In this paper, a dynamic strategy to represent the real world and the new COVID-19 disease is proposed. It mainly considers the impact of various measures taken by the government on the control of disease spreading. This paper considers the parameters impact of government investment, media publicity, medical treatment, and law enforcement on the rate of disease spreading. The parameters are set as mi, i = 1, 2, 3, 4, respectively. Thus, the rate of disease spreading can be written as:

$$m=\sum_{i=1}^{4}k_{i}m_{i}$$ (3)

where k_i_ is the weight of each measure.

The GA is mainly used to optimize parameters of the model, it can be divided as two parts. The first part describes that using the GA to optimize the conditions of city lockdown. First initialization model, set the initial value, with acceptable mortality as a condition of judgment. According to data analysis and relevant government regulations, a three-day mortality rate greater than 0.045 is defined as an unacceptable mortality rate. When the unacceptable mortality rate is reached, we will update and save the neural network parameters as a basis value for city lockdown.

$$unacceptable\;mortality\;rate=\frac{\lambda \alpha m\gamma }{\alpha \gamma (\lambda +m)+m\gamma ((\lambda +\beta )+\alpha \beta m}\geq 0.045$$ (4)

At the same time, further to run the model, with the minimum mortality as fitness function and update neural network parameters by genetic algorithm.

$$minimum\;mortality=\frac{\lambda \alpha m\gamma }{\alpha \gamma (\lambda +m)+m\gamma ((\lambda +\beta )+\alpha \beta m}$$ (5)

**Fig 3:**
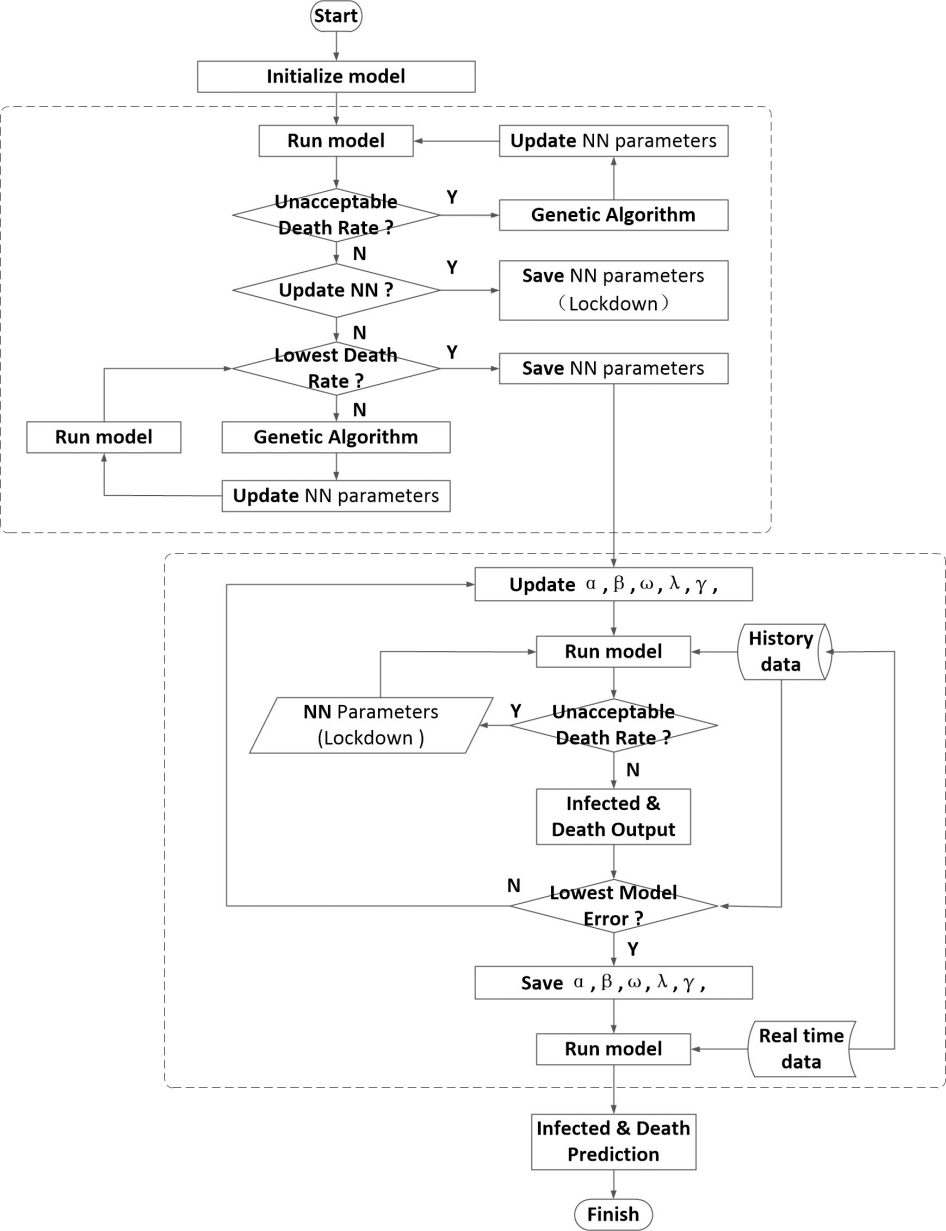
The flow chart of COVID-19 prediction algorithm.

Using twice genetic algorithm, the optimized neural network parameters is obtained, that is, the control measures. Further, the LSTM algorithm is used to modify other parameters of the model, and the historical data is compared with the data calculated by the model, such as the number of infections and deaths, to determine whether the system has reached the minimum error. The number of infections and deaths are further predicted. The flow chart of COVID-19 prediction algorithm is shown in Fig 3.

New data on the daily increase in the number of infected cases can be obtained. Using the same method, it can process the number of new deaths per day.

$$\{\begin{array}{c} \Delta I(t)=I(t)‐I(t‐1)\\ \Delta D(t)=D(t)‐D(t‐1) \end{array}$$ (6)

Here, I(t) is the cumulative number of infected cases in the previous t-day, I(t-1) is the cumulative number of infected cases in the previous t-day, and ΔI is the new number of infected cases in the first t-day. D(t) is the cumulative number of deaths in the first t-day, D(t-1) is the cumulative number of deaths in the first t-day, and *ΔD* is the additional number of deaths on the first t-day.

**Fig 4:**
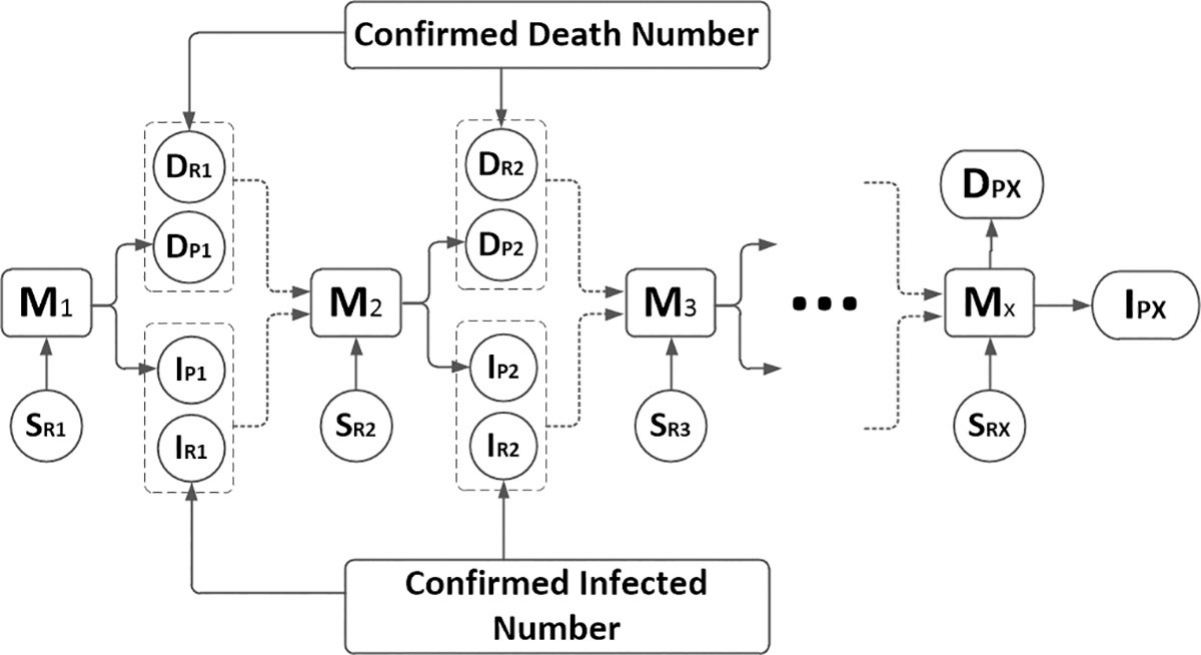
The LSTM method.

In order to get an accurate model, the model parameters need to be further processed. The GA has optimized the weight parameters in the neural network. Other model parameters are optimized through the optimized neural network model. Here, LSTM neural network is used to optimize the model parameters. We collected historical data (from February 26 to October 13 in Brazil) from the World Health Organization (WHO) on 15th October 2020. The data can be found in https://covid19.who.int/region/amro/country/br and contains three information types including “Date”, “Daily new confirmed” and “Daily new death”. Then the data was used to train neural network. Here we discussed the model parameters through two judgments. First, when the mortality rate of the model reaches an unacceptable mortality rate, the neural network parameters of the closed city are used to process the system model parameters, and then the mortality rate and infection rate are further compared with the actual data. By obtaining the minimum model error, the optimized model parameters are finally obtained. Here, let the actual infected rate be ΔI and the infected rate under the regression exponential function be ΔI’, and use the neural network to predict the deviation between the actual mortality and the regressive infection rate. Similarly, the same approach can be applied to mortality and obtained ΔD and ΔD’. Assuming that B = ΔI’+ΔD’ is the deviation characteristic of the prediction, the LSTM method can be used to predict the model. The flow diagram of the prediction is shown in Fig 4.

S_R1_ is the number of vulnerable infected persons on the first day, I_R1_ is the number of real-time infected cases on the first day, I_P1_ is the number of infected cases predicted on the first day, M_1_ is the initial model. Where S_RX_ is the number of vulnerable infected persons on day X, I_RX_ is the number of real-time infected cases on day X, I_PX_ is the predicted number of infected cases on day X, and M_X_ is the model on Day X.

## Simulation results and discussion

Based on GA in Fig 1, we computed the vulnerability coefficient k_i_ of four government measures. Without city lockdown, the infection probability m of government investment, media publicity, medical treatment and law enforcement are 0.174, 0.717, 0.021 and 0.853. With city lockdown, the infection probability m of government investment, media publicity, medical treatment and law enforcement are 0.085, 0.219, 0.107 and 0.349. There are two significant government measures media publicity and law enforcement, and the law enforcement is more important. Make a globally observation, each country invested huge amounts of money for COVID-19, and most medical workers tried their best to save lives. The difference of epidemic prevention results comes from the media publicity and law enforcement. Enhancing the influence of media can build a strong basis of epidemic prevention by offering the meaning of prevention measures. Considering the exception of inconsiderate and non-media audiences, the risk of disease spreading still exist. The efficiency of law enforcement is the crucial insurance to save the effort of medical workers.

**Fig 5:**
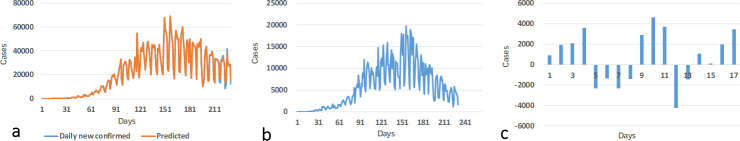
Brazil infected cases. a) Infection of predicted and confirmed per days, b) Infection prediction with best government performance, c) Infection prediction error.

We simulated the disease development and analyze the output of the model in 231 days with real epidemic data available from Brazil (February 26 to October 13). Fig 5A represents the number of predicted and confirmed per days, the model predicts infected per days of the last 17 days before October 13. Considering the lockdown from March 21 to July 31, the government realized the necessity of quarantine. From the data trend of infection confirmed per days over this period, the law enforcement shows insufficient efficiency. Supposing the government gave the best performance to control the disease, we performed the simulation about the development of infection and death per days. Fig 5B is the infected prediction with best performance of government; the possible infected number would much lower with effective government effort. With the comparison between Fig 5A and 5B, it is obvious that there is a great impact on the prediction in the middle and later period due to the large changes in the data after government make best moves. Fig 5C is the daily-infected prediction error of model for last 17 days. Considering the enormousness of infection confirmed number, Fig 5C shows the accuracy of model prediction without best performance of government.

**Fig 6:**
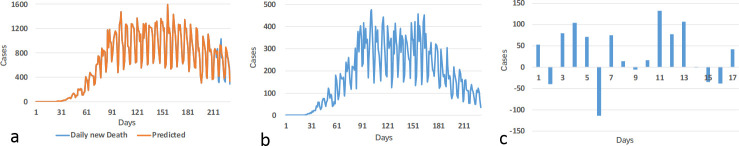
Brazil death cases. a) Death of prediction and confirmed per days, b) Death prediction with best government performance, c) Death prediction error.

To better understand the model performance, we simulated the number of deaths in Brazil. Fig 6A represents the number of new death cases per day, this indicates that at the beginning of the epidemic, due to limited understanding of disease transmission and limited detection efforts, there will be omissions in disease statistics, leading to a similar increase trend with the infected cases. Meanwhile, Fig 6A represents the new predicted death cases per days of the last 17 days before October 13, which shows the difference with daily new death cases. Fig 6B is the daily death prediction with best performance of government. While the continuous improvement of detection methods and the continuous promotion of detection scope will be carried out, the government could strengthen the control of the disease, so the daily death cases would gradually decrease. Fig 6C is the daily death prediction error of model for last 17 days. Combine Fig 6A, we can see that the LSTM method have a good performance to predict COVID-19 disease. The method can predict the trend of the disease over a longer period with the performance of government.

## Conclusions

Based on the SIQR epidemic spreading model, this paper seeks the best government performance from the four aspects by GA; and then proposed a hybrid prediction model with LSTM. By analyzing the new confirmed cases and death rate of Brazil data from February 26 to October 13, it is found that media publicity and law enforcement have more contribution to reduce transmission rate. With best government performance, the trend of COVID-19 in Brazil could under control. The prediction results of this model are highly consistent with the actual epidemic situation, which proves that the hybrid model proposed in this paper can efficiently analyze the transmission law and development trend of the virus. So, modeling the minimum mortality rate would be of the utmost importance for nations to prevent and control COVID-19. For future research, the epidemic spreading on multi-layers networks and hybrid intelligent algorithm is worthy of considering.

## Supporting information

S1 Dataset (ZIP)
