# Correction: Prediction and control of COVID-19 spreading based on a hybrid intelligent model

**DOI:** 10.1371/journal.pone.0306723

**Published:** 2024-07-03

**Authors:** Gengpei Zhang, Xiongding Liu

There is an error in affiliation 1 for author Gengpei Zhang. Affiliation 1 should be: School of Electronic Information and Electrical Engineering, Yangtze University, Jingzhou City, Hubei Province, China
